# Analysis of the *FBXO7* promoter reveals overlapping Pax5 and c-Myb binding sites functioning in B cells

**DOI:** 10.1016/j.bbrc.2021.03.052

**Published:** 2021-05-21

**Authors:** Rebecca Harris, Suzanne Randle, Heike Laman

**Affiliations:** University of Cambridge, Department of Pathology, Tennis Court Road, Cambridge, CB2 1QP, United Kingdom

**Keywords:** Fbxo7, Pax5, c-Myb, ELF4, Transcription factor, Promoter, B cells

## Abstract

Fbxo7 is a key player in the differentiation and function of numerous blood cell types, and in neurons, oligodendrocytes and spermatocytes. In an effort to gain insight into the physiological and pathological settings where Fbxo7 is likely to play a key role, we sought to define the transcription factors which direct *FBXO7* expression. Using sequence alignments across 28 species, we defined the human *FBXO7* promoter and found that it contains two conserved regions enriched for multiple transcription factor binding sites. Many of these have roles in either neuronal or haematopoietic development. Using various *FBXO7* promoter reporters, we found ELF4, Pax5 and c-Myb have functional binding sites that activate transcription. We find endogenous Pax5 is bound to the *FBXO7* promoter in pre-B cells, and that the exogenous expression of Pax5 represses Fbxo7 transcription in early pro-B cells.

## Highlights

• We defined the human *FBXO7* promoter, as a conserved promoter region between −1300 and + 100 bp from the start of exon 1.
• Two conserved islands of putative transcription factor binding sites were found with 32 putative binding sites identified for 24 different TFs (17 in the distal region; 15 in the proximal region).
• ETS factors, ELF4 and ELF1, and Pax5 and c-Myb bind and activate *FBXO7* luciferase reporter constructs.
• Fbxo7 represses transcription from its own promoter, and this is a ubiquitin ligase-independent effect.

## Introduction

F-box proteins (FBP) are exchangeable subunits within Skp1-Cullin1-F-box protein (SCF)-type E3 ubiquitin ligases. These enzymes conjugate a 76aa ubiquitin peptide onto proteins, and this post-translational modification can precipitate that target’s degradation, change of localisation or activity. Fbxo7 is one of ∼70 F-box domain-containing proteins, which are receptors for SCF-E3 ligases. However, Fbxo7 also functions outside of canonical ubiquitin-dependent pathways, for example, acting as scaffolds for other regulatory proteins [1]. At a physiological level, mis-regulation of Fbxo7 has been implicated in human diseases with disparate aetiologies, including neurological diseases, anaemia and cancer, attesting to its pleiotropic role in numerous cell types [[2], [3], [4]].

Fbxo7 has hundreds of potential substrates making the discovery of the critical pathways affected by its mis-regulation in pathological settings very challenging [5]. At a molecular level, Fbxo7 affects many processes including, the cell cycle, enhancing cyclin D/Cdk6 activity by acting as a scaffold for their assembly and stabilising the cyclin-dependent kinase inhibitor, p27 [6]; the regulation of stress-induced mitophagy via the PINK1/Parkin pathway [7]; NF-κB signalling [[8], [9], [10]], and BMP signalling via NRAGE-TAK1-TAB1 complex formation [11]. In addition, Fbxo7 ubiquitinates proteasomal subunits, like PSMA2, affecting the assembly of proteasomes, ribosomal subunits, like stress-responsive subunit RPL23, to induce p53 transcriptional responses, and most recently the kinase, PINK1 [5,[12], [13], [14], [15], [16], [17]]. In addition, Fbxo7 is essential for male fertility, and this is attributed to the stabilisation of a proteasomal regulator and trafficker, PI31 [[18], [19], [20], [21], [22]]. The severity of phenotypes in neurons, erythrocytes, spermatocytes and lymphocytes demonstrate the essentialness of Fbxo7-regulated pathways.

We and others have reported the important role of Fbxo7 in the development and differentiation of B and T lymphocytes [[22], [23], [24]]. In addition, Fbxo7 can be oncogenic as its over-expression on a background of p53 mutation promotes T cell lymphomagenesis [25]. Recently, a number of high-resolution studies of transcription factor (TF) networks reveal the TFs that function in the normal expansion and differentiation of immature progenitor cells are often dysregulated in leukaemia and lymphoma [[26], [27], [28], [29], [30]]. Since Fbxo7 and its proto-oncogenic partners, CDK6 and cyclin D2 and D3 are key cell cycle regulators in B and T cells, we investigated whether any of the lineage-specifying haematopoietic TFs control the transcription of Fbxo7. We sought to identify potential TF binding sites within the *FBXO7* promoter and to investigate their regulation of Fbxo7 expression. We identified several promoter elements for TFs, including ELF4, c-Myb and Pax5, which is a master regulator of B cell differentiation, neural development, and spermatogenesis.

## Materials and methods

***Promoter alignment***. Transcription start sites (TSS) within the NCBI RefSeq *FBXO7* gene sequence were identified using Eponine software. Fbxo7 orthologues from 28 mammalian species were aligned using the USCS Comparative Genomics 28-way vertebrate alignment and conservation track, and regions of conservation identified. Sequences 10 kb upstream to 10 kb downstream of the gene start site were analysed until conserved regions of similarity stopped. Putative conserved TF binding sites were then identified using MatInspector software. Sites found in more than half of the species were annotated. Data was presented in Clustal W format. This analysis was performed by Dr Michael Mitchell of the CRUK Bioinformatics & Biostatistics Service.

**FBXO7 *promoter cloning.*** A 1.7 kb DNA region containing sequences approximately 1.3 kb upstream to 0.4 kb downstream of the *FBXO7* TSS was amplified by PCR from genomic DNA, and subcloned into pGEM-T Easy vector (Promega). Luciferase reporter constructs were amplified from this plasmid, including a 1.5 kb region (containing the full length *FBXO7* promoter, termed Fbxo7-luc), a 0.5 kb proximal promoter region (proximal Fbxo7-luc), and a 0.6 kb distal promoter region (distal Fbxo7-luc), which were sub-cloned into pTA-luc (Clontech). Site-directed mutagenesis was used to mutate the Pax5 and ETS binding sites. All mutations were verified by sequencing.

***Cell culture and biochemistry.*** U2OS, Eco Phoenix cells and B cell lines (Nalm6, Ba/F3, Raji, A20) were maintained as in Ref. [23]. Immunoblotting was performed as in Ref. [3].

***Luciferase assay.*** U20S cells were transfected with 200 ng of reporter plasmid DNA, 200 ng of pEF-LacZ, and 200 ng of mammalian expression vectors. After 48 h cells were harvested, and luciferase assays performed as in Ref. [5].

***RT-qPCR****.* Experiments were performed as in Ref. [3]. Expression was normalised to cyclophilin levels, and values expressed relative to vector control cells.

***Chromatin immunoprecipitation.*** Chromatin immunoprecipitation (ChIP) was performed as recommended using the ChIP-IT Express kit (Active Motif). 100 μL of sheared chromatin was immunoprecipitated with 3 μg anti-Pax5, IgG or anti-RNA polymerase II (Human ChIP-IT control kit; Active Motif) and precipitated by magnetic protein G beads.

***Semi-quantitative and quantitative PCR.*** PCR primer pairs encompassed the putative Pax5 binding site in *FBXO7*, and that previously published for the CD19 promoter [31], as well as negative control GAPDH primers supplied in the ChIP-IT control kit. Primer pairs were used in triplicate PCR reactions and performed as in Ref. [3].

***Expression constructs.*** Pax5 and ELF4 cDNAs were sub-cloned in frame to FLAG or T7 epitope tags in pcDNA3 (Invitrogen). A Pax5 expression construct, pX-13, was provided by Dr F Baumann-Kubetzko**.** Full length pCB6-MEF with ELF4 sequences was provided by Dr MA Suico. A Pax5 retroviral construct was generated by sub-cloning into MCSV-IRES-GFP. Pax5 shRNA sequences targeting endogenous mouse Pax5 mRNA were from the RNAi Codex database (codex.cshl.edu). shRNA vectors were cloned into MSCV-LTRmiR30-IRES-GFP. All vectors were verified by sequencing.

***Antibodies and primers*** used in this study are listed in Supplementary Information.

## Results

### The *FBXO7* promoter contains two TF islands

**Fig. 1:**
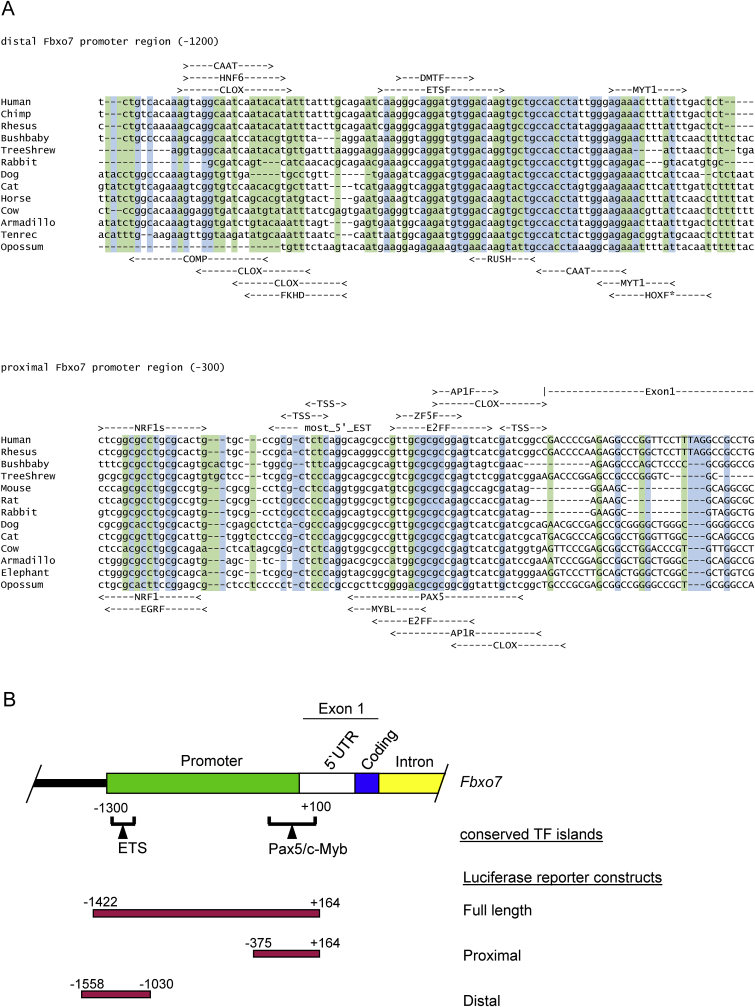
Sequence alignment of 13 mammalian species (**A**, upper) showing −1275 to −1176 of the distal TF island which covers −1275 to −1151, and (**A**, lower) from −66 to +35 of the proximal TF island which covers −300 to +100, numbered according to the human sequence from the start of exon 1. Putative TF binding sites are listed above the alignment on the forward strand, and below the alignment on the reverse strand. Conserved bases are shown in blue and conserved pyrimidines or purines, in green. (**B**) Schematic of the start of human *FBXO7*. The promoter (green) was defined by sequence alignment of several species (see M&M). Several TF binding sites were identified within conserved TF islands (brackets). The 5′ UTR (white), coding sequences (blue), and first intron (yellow) are indicated. Luciferase reporters (red) are below. (For interpretation of the references to colour in this figure legend, the reader is referred to the Web version of this article.)

To investigate the transcriptional regulation of *FBXO7*, its promoter was analysed for the presence of TF binding sites. Sequence alignment of 28 mammalian species identified a region of conserved sequence similarity upstream of the *FBXO7* transcription start sites (TSS). Consequently, the *FBXO7* promoter was delineated as starting 1300bp upstream from the start of exon 1, to 100bp downstream, numbered according to the human *FBXO7* gene (−1300 to +100). Within this region, two clusters of conserved TF binding sites were identified, approximately 1 kb apart. One island in the distal promoter region was 125bp in length (−1275 to −1150), while the other in the proximal promoter region was 400bp in length and overlapped the TSS and start of exon 1 (−300 to +100). These islands contained the majority of TF binding sites, and selected regions from 13 of the species surveyed are shown in Fig. 1A. 32 putative binding sites were identified for 24 different TFs (17 in the distal region; 15 in the proximal region). We identified ‘core’ promoter elements, including two CCAAT boxes on opposite strands at −1257 and −1198 in the distal region, and a GC box, identified as an Sp1 binding site, in the proximal region within exon 1 (+81). No TATA box was identified, and the proximal region was GC rich, suggesting the *FBXO7* promoter is associated with a CpG island. In addition to ubiquitously expressed TFs, like activator protein (AP) and E2Fs, we identified multiple consensus sites for TFs with roles in haematopoiesis and neuronal development (ETS, c-Myb, DMTF, Pax5, Myt1, NeuroD, NRF1, CLOX, ZF5F).

### The FBXO7 reporter is activated by ETS factors, including ELF4

**Fig. 2:**
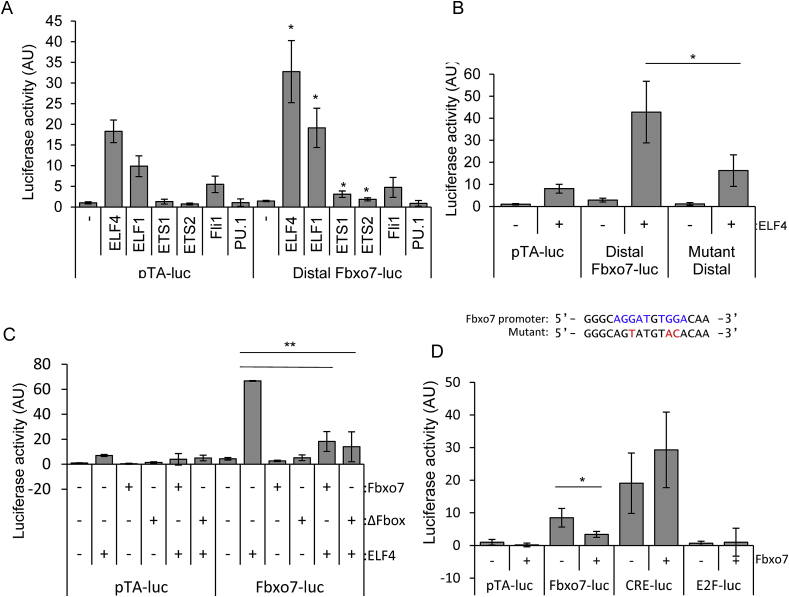
(**A**) Luciferase assay of cell lysates from U2OS cells transfected with distal Fbxo7-luc, or pTA-luc empty vector, along with a panel of six ETS family members. Luciferase values in triplicate, were background corrected with non-transfected cell lysate values, and normalised to co-transfected β-galactosidase levels, and expressed relative to empty vector (−). ∗*p* < 0.05 compared to the relevant empty control levels, *n* = 3. (**B**) Luciferase assay showing ELF4 activation of the empty vector, distal Fbxo7-luc and mutated distal Fbxo7-luc reporter constructs, *n* = 3. Below: the two ELF4 consensus sites in the *FBXO7* promoter are shown in blue, and mutated base pairs in red. (**C**) Luciferase assays showing ELF4 activity on the Fbxo7-luc reporter in the presence of exogenous Fbxo7 or ligase-dead mutant Fbxo7-ΔFbox. (**D**) Luciferase assays in cells transfected with a panel of luciferase reporters, with or without Fbxo7, *n* = 3. (For interpretation of the references to colour in this figure legend, the reader is referred to the Web version of this article.)

To test putative binding sites, *FBXO7* reporters were generated. Full length *FBXO7* promoter (−1422 to +164), as well as either the distal (−1558 to −1030) or proximal (−375 to +164) regions were cloned into pTA-luc luciferase plasmid (Fig. 1B). Within the distal region, we identified two ETS binding sites (EBS), shown in Fig. 1A as ETSF. The consensus site contains a core GGAA sequence, but flanking sequences and co-factor binding impart specificity for particular ETS factors. We tested a panel of ETS factors (ETS1, ETS2, Fli1, ELF4, ELF1, and PU.1) by co-transfecting them with the distal reporter (Distal Fbxo7-luc) or a control (pTA-luc) into U20S cells (Fig. 2A). The transfection of ELF4, ELF1 and Fli1 increased transcription of the pTA-luc control reporter, indicating the presence of cryptic response elements for three of the six ETS factors tested. However, transfection of this panel of ETS factors with the distal Fbxo7-luc reporter showed that ELF4 and ELF1, and also ETS1 and ETS2, and significantly increased activation of the reporter (*p* < 0.05), above the background level of activation seen in the control reporter. These data suggest the presence of functional EBSs in the distal promoter. We noted both EBSs contain a WGGA (where W is A/T) sequence, which matches the consensus site for ELF4, consistent with its higher activation of the reporter. Mutation of the EBSs significantly reduced ELF4 transactivation (*p* < 0.05) (Fig. 2B), indicating that ELF4 utilised these sites to activate the distal reporter.

### Fbxo7 inhibits ELF4 trans-activation

Lui and co-workers reported Fbxo7 and ELF4 physically interact [32], which suggests Fbxo7 may affect ELF4 transactivation. To test this, ELF4 was co-transfected with either WT Fbxo7 or a mutant lacking the F-box domain (ΔF-box), and the full-length luciferase reporter into U2OS cells. ELF4 activation of the reporter was inhibited 80% by the addition of WT or mutant Fbxo7 (Fig. 2C), indicating Fbxo7 inhibits ELF4 transactivation and does so in a ubiquitin-independent manner. We next tested whether this effect was dependent on ELF4 or a general effect on *FBXO7* and other reporters, by transfecting Fbxo7 and measuring luciferase expression from other reporters. As before, we found Fbxo7 significantly inhibited the full length Fbxo7-luc, but not the Cre-luc or E2F-luc reporters (Fig. 2D). Moreover, as U20S cells do not express ELF4, these results suggests that Fbxo7 may bind to other ETS factors. Together, these data suggest Fbxo7 can inhibit transcription from its own promoter in an SCF ligase-independent manner.

### An FBXO7 gene reporter is activated by Pax5 and c-Myb

**Fig. 3:**
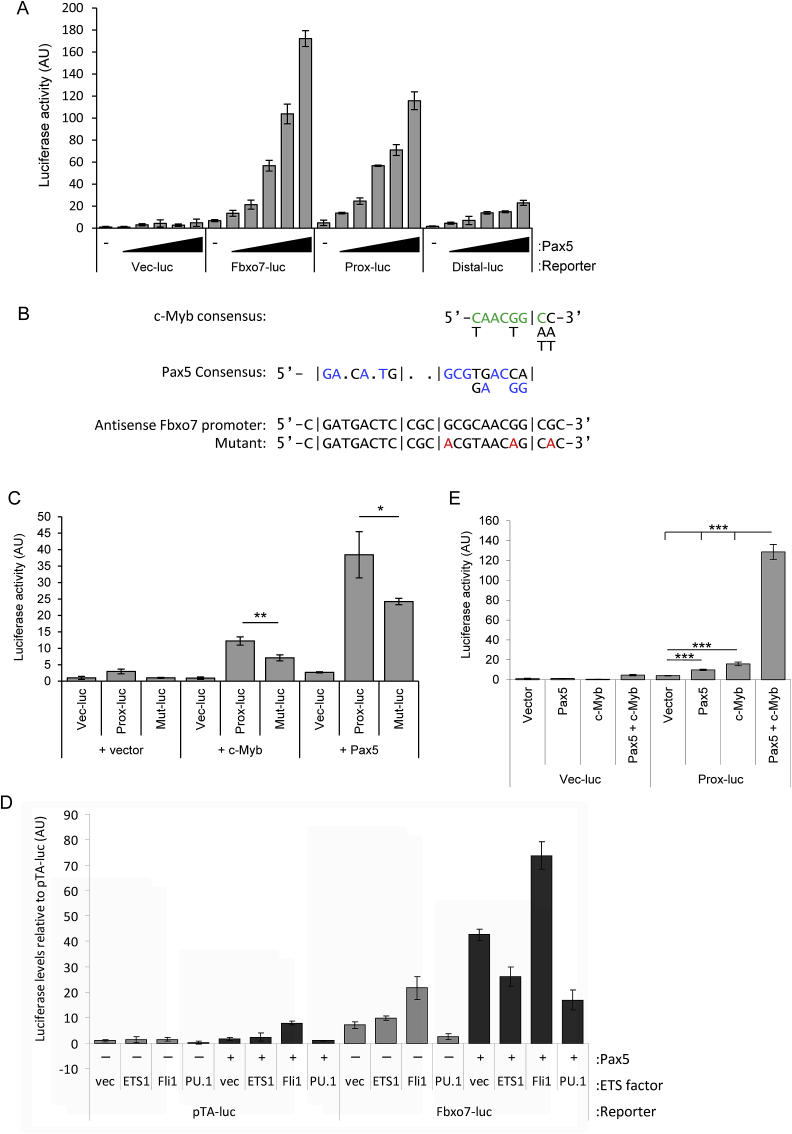
(**A**) Luciferase assay with various *FBXO7* reporters and increasing doses of Pax5. (**B**) A c-Myb binding site overlaps a Pax5 binding site. Shown are the Pax5 binding site (blue), with bases in the *FBXO7* promoter matching the consensus for c-Myb (green), and the mutated bases in the Proximal *FBXO7* reporter (red). (**C**) Luciferase assay on U2OS cell lysates co-transfected with Pax5 or c-Myb, and the empty, WT, or mutated proximal Fbxo7-luc reporters. (**D**) Luciferase assay showing activation of various Fbxo7 luciferase vectors by vector only (vec) or exogenous ETS family members, with (+, dark grey) or without (-, light grey) Pax5. **(E)** Luciferase assay on U2OS cell lysates co-transfected with Pax5 or c-Myb, in combination, with the proximal Fbxo7-luc reporter. (For interpretation of the references to colour in this figure legend, the reader is referred to the Web version of this article.)

ETS factors act in concert with other TFs, like Pax5. We identified a Pax5 consensus site in the proximal region. To test its functionality, U20S cells were co-transfected with a Pax5 expression construct or a control and the reporters, and luciferase assays were performed. A dose-dependent increase in luciferase activity was detected with increasing Pax5 expression from the full length and proximal *FBXO7* constructs (Fig. 3A), with a more limited response from the distal reporter. This suggests that Pax5 activation of the full-length promoter is predominantly via the proximal region. We also mutated the Pax5 binding site within the proximal reporter (Fig. 3B) and tested the effect (Fig. 3C). Expression of Pax5 resulted in a 40-fold induction of luciferase activity from the proximal reporter, whereas Pax5 activation of the mutant reporter was significantly reduced by 37% (Fig. 3C). These data argue Pax5 activation of the reporter occurs at the Pax5 binding site. We next tested whether Pax5 activation was modulated by ETS family members. As in Fig. 3A, Pax5 activated the full-length reporter (Fig. 3D). However, when Fli1 was co-transfected with Pax5, luciferase levels increased by 70% over Pax5 alone, and 340% more than Fli1 alone. In contrast, addition of ETS1 or PU.1 inhibited Pax5 activation by 40% and 60%, respectively.

A c-Myb binding site overlaps the Pax5 binding site (Fig. 1A), and c-Myb has also been shown to influence Pax5 activity [33,34]. To test the c-Myb binding site, we transfected c-Myb along with either the WT or mutated proximal reporter constructs (Fig. 3B) and found c-Myb activated the proximal region by 12-fold (Fig. 3C). Furthermore, the mutated reporter, which contained one altered base pair within the consensus c-Myb site, showed decreased activation with c-Myb by 40% compared to the WT promoter. As Pax5 and c-Myb both activated the proximal reporter through an overlapping binding site, we tested whether they had a synergistic or competitive effect on transactivation. When transfected together, Pax5 and c-Myb activated the proximal reporter more than 8-fold over c-Myb alone, and over 13-fold than Pax5 alone (Fig. 3E), suggesting they act synergistically through this site. These data indicate that Pax5 together with ETS and c-Myb TFs transactivate the *FBXO7* reporters.

### Endogenous Pax5 binds to the Fbxo7 promoter

**Fig. 4:**
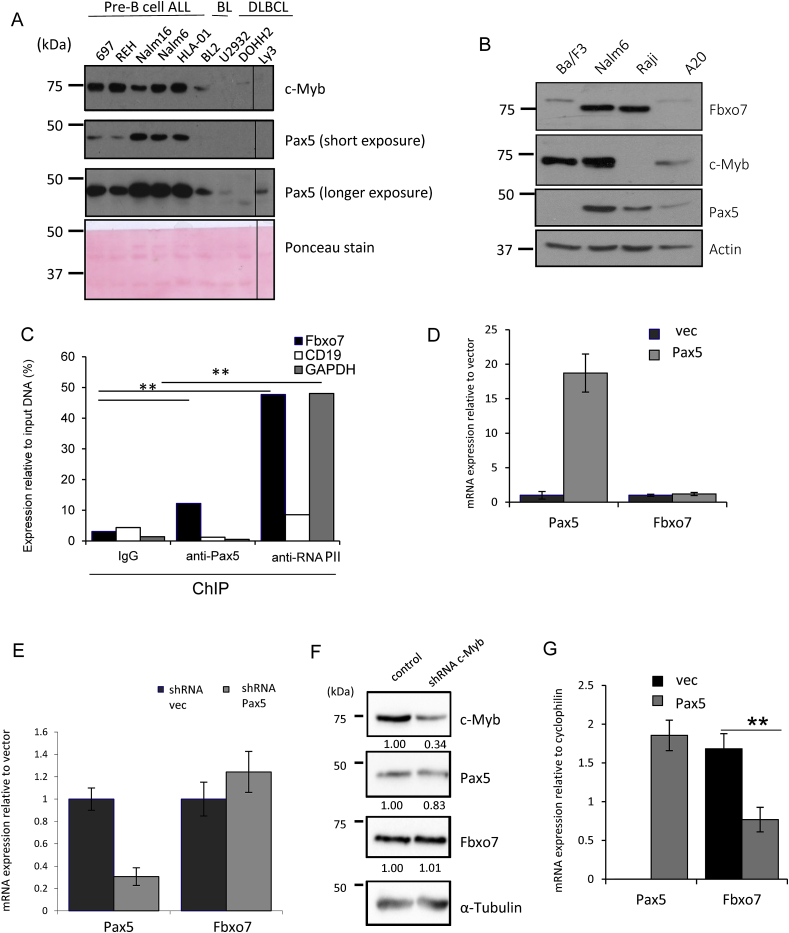
(**A**) Human B cell tumour lines analysed by immunoblotting for the expression of Pax5 and c-Myb. ALL = Acute lymphocytic leukaemia, BL = Burkitt’s lymphoma, DLBCL = diffuse large B cell lymphoma. (**B**) Immunoblotting of cell lysates from murine Ba/F3 pro-B cell, pre-B cell, and mature B cell lines for Fbxo7, Pax5 and c-Myb expression. (**C**) Pax5 chromatin immunoprecipitation of the *FBXO7* promoter in Nalm6 cells. Values were then expressed as a percentage relative to input. ∗∗*p* < 0.01 compared to relevant control IgG levels. *n* = 2 independent experiments with triplicate qPCR reactions. (D) Graphs of mRNA expression of Nalm6 cells transduced to over-express Pax5 (grey bars) or empty vector (black bars), *n* = 2. (**E**) Graph of mRNA expression in Nalm6 cells transduced to express a shRNA construct targeting Pax5 (grey bars), or empty vector (black bars). Pax5 and Fbxo7 mRNA levels were analysed by RT-qPCR, normalised to cyclophilin A levels and expressed relative to empty vector control cells. (**F**) Immunoblotting of Nalm6 cell lysates transduced with shRNA constructs targeting c-Myb expression, *n* = 2. **(G)** Graphs of mRNA expression of Ba/F3 cells transduced to over-express Pax5 (grey bars) or empty vector (black bars), *n* = 2.

As Pax5 and c-Myb are both involved in B cell development, a screen of c-Myb and Pax5 protein expression was conducted in a panel of B cell tumour lines originating from B cells at different stages of maturation (Fig. 4**A**). Both proteins were noted to be expressed in the earlier stages of B cell development, and their expression decreased as B cells matured, consistent with roles in B cell maturation. Immunoblotting for Fbxo7 expression, we noted that Fbxo7 correlated with Pax5, but not necessarily c-Myb. For example, Fbxo7 expression was readily detected in Nalm6 and Raji cell lysates, but Raji cells had no detectable c-Myb expression (Fig. 4B).

We next tested whether Pax5 was present at the *Fbxo7* promoter in maturing B cells, using chromatin immunoprecipitation (ChIP) assays. The *CD19* promoter, a Pax5 target, and *GAPDH*, which does not contain a consensus Pax5 site, were selected as promoter controls, and antibodies to RNA polymerase II and normal serum IgG were used as immunoprecipitation controls. ChIP experiments were performed in Nalm6 cells, which showed high expression of Pax5, Fbxo7, and c-Myb. Immunoprecipitated DNA was analysed by qPCR, and DNA enrichment expressed as a percentage of input DNA to normalize for differences in PCR efficiency. The *FBXO7* promoter was enriched 4-fold in DNA immunoprecipitated using Pax5 antibody compared to IgG only, and 32-fold over the amount of *GAPDH* promoter region immunoprecipitated by Pax5 antibody (Fig. 4C). This was despite a significant enrichment of the *GAPDH* promoter by RNA polymerase II antibodies. Enrichment of the *FBXO7* promoter in Pax5 immunoprecipitates, and enrichment of the *FBXO7* and *GAPDH* promoter in RNA polymerase II immunoprecipitates were significantly increased (*p* < 0.01) compared to IgG levels. Unexpectedly, there was no significant enrichment of the *CD19* promoter in Pax5 or RNA polymerase II immunoprecipitates, suggesting CD19 was not transcribed. We confirmed the lack of CD19 expression by flow cytometry of the cells with a fluorescently labelled anti-CD19 antibody (data not shown). Similar enrichment of the *FBXO7* promoter in Pax5 immunoprecipitates were found in murine A20 cells. These data indicate the Pax5 binding site in the *FBXO7* promoter is a functional TF binding site in B cells and suggest Pax5 directly regulates *FBXO7* expression.

### Early expression of Pax5 reduced Fbxo7 expression in pro-B cells but not in later stage B cells

We then performed experiments to alter the expression of Pax5 in Nalm6 cells, which expressed Pax5, c-Myb and Fbxo7. Nalm6 cells were transduced with retroviruses encoding Pax5 to further increase its expression; however, no changes in Fbxo7 mRNA levels were observed (Fig. 4D). Similar negative results were seen in A20 cells (data not shown). We also tested the effect of reducing Pax5 expression using a short hairpin construct in Nalm6 cells. The shRNA caused an approximate 70% reduction in Pax5 mRNA levels, and although there was a small increase in Fbxo7 expression, this was not a statistically significant change (Fig. 4E). We considered whether c-Myb might regulate Fbxo7 expression in Nalm6 cells, so we also targeted its expression using a shRNA against c-Myb and expressing GFP. Cells were transduced with viruses expressing shRNAs and then selected and cloned by limiting dilution. Cell lysates were assayed by immunoblotting (Fig. 4F). Surprisingly, reduction of c-Myb expression also reduced Pax5 expression, suggesting c-Myb stabilises Pax5 expression; however, despite reductions in the levels of both transcription factors, there was no effect on Fbxo7 levels in Nalm6 cells.

We tested whether Pax5 would influence Fbxo7 transcription in earlier stage B cells lacking endogenous Pax5 expression. Ba/F3 pro-B cells were infected with retroviruses encoding Pax5 and GFP. Total RNA was harvested from GFP + ve cells, and mRNA levels were assayed using RT-qPCR. We observed a 50% reduction in Fbxo7 mRNA levels when Pax5 expression was introduced (Fig. 4G). Thus, ectopic expression of Pax5 in a pro-B cell affected Fbxo7 expression, while over-expression in a pre-B cell did not. Our data are consistent with ectopic Pax5 binding at the endogenous *FBXO7* promoter and repressing transcription.

## Discussion

To investigate Fbxo7 transcriptional regulation, we defined the human *FBXO7* promoter, as a conserved promoter region between −1300 and + 100 bp from the start of exon 1. Within the promoter two conserved TF islands (−300 to +100 and −1275 to −1150) were identified. A similar proximal promoter was reported in the pig, although it was limited to 1000 bp upstream of the TSS [35]. Although no TATA box was identified, as for the pig, the CCAAT and GC boxes may constitute part of the core promoter of *FBXO7*. Consistent with this, analysis of the publicly available gene annotation database Encyclopaedia of DNA Elements (ENCODE; http://genome.ucsc.edu/ENCODE/) [36], suggests that the proximal region, along with exon 1 of *FBXO7* lies within a CpG island, H3K4 tri-methylation region and DNase I hypersensitivity site, indicating these sequence form the core *FBXO7* promoter.

Given the key roles of the Fbxo7 in blood, we investigated TF binding sites with known roles in haematopoiesis. These included twin ETS binding sites in the distal region of *FBXO7* and overlapping Pax5 and c-Myb sites in the proximal region. We found Pax5, c-Myb and the ETS family members, ELF4 and ELF1, were the strongest activators of a synthetic *FBXO7* reporter. Interestingly, Fbxo7 has been previously reported to directly interact with ELF4. Our data indicates that Fbxo7 inhibits ELF4 activation of the *FBXO7* reporter, an activity independent of its ubiquitin ligase function, suggesting a negative feedback mechanism. Also, since Fbxo7 did not inhibit other luciferase reporters, this suggests Fbxo7 specifically inhibits its own transcription.

The *FBXO7* reporter was also transactivated by Pax5, whose expression is largely restricted to B cells. Using ChIP assays, we found that endogenous Pax5 was bound to the proximal region of *FBXO7* in both the mature B cell line, A20, and a leukaemic pre-B cell line, Nalm6, indicating a functional Pax5 binding site. Despite this, only the exogenous expression of Pax5 in Ba/F3 cells caused a change in *FBXO7* mRNA expression, repressing transcription. We believe this difference between reporter assays and endogenous transcription reflects the chromatin nature of transcriptional activation. It is known that Pax5 acts in concert with other TFs such as ETS factors [33,37]. Pax5 recruits ETS factors, Net and Elk-1, to the *mb-1* promoter in pre-B cells to increase DNA binding, whereas PU.1 recruits Grg4 to Pax5-occupied promoters where Pax5 represses transcription [37]. We found several ETS factors modulated Pax5 transactivation of an *FBXO7* reporter, including Fli1 which increased transcription, and ETS1 and PU.1 which inhibited it. Interestingly, a study of TF networks in haematopoietic cells using combined data from 53 ChIP-seq studies, identified PU.1 at the *FBXO7* promoter, highlighting that PU.1 also regulates *FBXO7* expression [38]. Pax5 also enlists other TFs like c-Myb, e.g., Pax5 recruits c-Myb to the *RAG-2* promoter in B cells [33], and our data indicate c-Myb may affect Pax5 levels and thus indirectly affect transcription at the *FBXO7* promoter.

Although Pax5, c-Myb, ETS, have important roles in haematopoiesis, c-Myb and Pax5 also have roles in neural progenitor cell proliferation [39], and early midbrain development [40], respectively. Fbxo7 is expressed in mouse adult brain [15], so whether these TF sites are active during brain development warrant further study. Intriguingly, given Fbxo7’s role in Parkinson’s disease, a site for nuclear respiratory factor 1 (NRF1) was also found in its promoter. This TF is associated with mitochondrial function and metabolism, neurite outgrowth, and the ‘bounce back’ of proteasome transcription under proteotoxic stress [[41], [42], [43], [44]]. That multiple TFs co-operate to regulate Fbxo7 expression and are involved both in cell differentiation and stress-responsive functions points to a model wherein the proteins that specify cellular lineages and mature cell types also have a role in maintaining their health when they come under stress.
