# Supplementary material for: Analysis of the FBXO7 promoter reveals overlapping Pax5 and c-Myb binding sites functioning in B cells
Source: Biochem Biophys Res Commun. 2021 May 21;554:41–8. doi: 10.1016/j.bbrc.2021.03.052 (PMC8082276; doi:10.1016/j.bbrc.2021.03.052)
Supplement: Multimedia component 1 [file mmc1.docx]

| **Primers** | **Forward (5’ to 3’)** | **Reverse (5’ to 3’)** |
| --- | --- | --- |
| FLAG-Pax5 | cctgagatctatggatttagagaaaaat | ccgctcgagtcagtgacggtcataggc |
| Pax5 shRNA template | tgctgttgacagtgagcgagcaggtattatgagacaggaatagtgaagccacagatgtattcctgtctcataatacctgcctgcctactgcctcgga | |
| ChIP mouse Fbxo7 | caagtgcgcccaagcaaacccag | gtcgactacgccccctagagcctt |
| ChIP mouse CD19 | ccctttcctctatacggggactg | ggcctgcctcctactaaggtaag |
| ChIP human Fbxo7 | cccagccaccgccgcggaaaatct | cggcaggcggcctaaaggaacc |
| ChIP human CD19 | cccgtggtagtgagagctgggatg | ggtggcatggtggtcagactctcc |
| RT-PCR mouse Fbxo7 | cgcagccaaagtgtacaaag | aggttcagtacttgccgtgtg |
| RT-PCR mouse Pax5 | aacccatcaagccagaacag | ggggaacctccaagaatcat |
| RT-PCR human Fbxo7 | ttccaggacctaaccccatctt | cgacccctgctgggtctaa |
| RT-PCR human Pax5 | ggaggagtgaatcagcttgg | ggcttgatgcttcctgtctc |
| RT-PCR cyclophilin | ccttgggccgcgtctcctt | caccctggcacatgaatcctg |

| **Antibody** | **Species** | **Dilution** | **Source** | **Catalog #** |
| --- | --- | --- | --- | --- |
| Actin | Rabbit | 1:5000 | Sigma | A2066 |
| Fbxo7 (Ab2) * | Rabbit | 1:500 | In house | C-terminus |
| Fbxo7 (Ab1) | Rabbit | 1:1000 | Aviva | ARP43128_P050 |
| Pax5 | Mouse | 1:500 | Santa Cruz | sc-55515 |
| c-myb | Mouse | 1:1000 | Millipore | 05-175 |
| Anti-Mouse | Goat | 1:10000 | Santa Cruz | sc-2055 |
| Anti-Rabbit | Donkey | 1:10000 | Santa Cruz | sc-2313 |
